# The Role of miRNA and Related Pathways in Pathophysiology of Uterine Fibroids—From Bench to Bedside

**DOI:** 10.3390/ijms21083016

**Published:** 2020-04-24

**Authors:** Michał Ciebiera, Marta Włodarczyk, Stanisław Zgliczyński, Tomasz Łoziński, Klaudia Walczak, Artur Czekierdowski

**Affiliations:** 1 Second Department of Obstetrics and Gynecology, The Center of Postgraduate Medical Education, 01-809 Warsaw, Poland; 2 Department of Biochemistry and Pharmacogenomics, Faculty of Pharmacy, Medical University of Warsaw, 02-097 Warsaw, Poland; marta.wlodarczyk@wum.edu.pl; 3 Center for Preclinical Research, Medical University of Warsaw, 02-097 Warsaw, Poland; 4 Department of Internal Diseases and Endocrinology, Central Teaching Clinical Hospital, Medical University of Warsaw, 02-097 Warsaw, Poland; stanislaw.zgliczynski@gmail.com; 5 Department of Obstetrics and Gynecology, Pro-Familia Hospital, 35-302 Rzeszów, Poland; tomasz-lozinski@pro-familia.pl; 6 Students’ Scientific Association at the Department of Endocrinology, The Center of Postgraduate Medical Education, 01-809 Warsaw, Poland; walczak.klaudia95@gmail.com; 7 Department of Gynecological Oncology and Gynecology, Medical University of Lublin, 20-081 Lublin, Poland; a.czekierdowski@umlub.pl

**Keywords:** uterine fibroid, uterine leiomyoma, biology, pathophysiology, microRNA, miRNA, non-coding RNA, diagnosis, treatment

## Abstract

Uterine fibroids (UFs) are the most common benign tumors of the female genital tract. Their prevalence usually is estimated at 30–40%, but may reach up to 70–80% in predisposed groups of women. UFs may cause various clinical issues which might constitute the major reason of the overall deterioration of the quality of life. The mechanisms leading to UFs formation and growth still remain poorly understood. The transformation of smooth muscle cells of the uterus into abnormal, immortal cells, capable of clonal division, is thought to be a starting point of all pathways leading to UF formation. Micro-ribonucleic acids (miRNAs) are non-coding single-stranded RNAs about 22 nucleotides in length, that regulate gene expression. One of recent advances in this field is the comprehension of the role of miRNAs in tumorigenesis. Alterations in the levels of miRNAs are related to the formation and growth of several tumors which show a distinct miRNA signature. The aim of this review is to summarize the current data about the role of miRNAs in the pathophysiology of UFs. We also discuss future directions in the miRNA research area with an emphasis on novel diagnostic opportunities or patient-tailored therapies. In our opinion data concerning the regulation of miRNA and its gene targets in the UFs are still insufficient in comparison with gynecological malignancies. The potential translational use of miRNA and derived technologies in the clinical care is at the early phase and needs far more evidence. However, it is one of the main areas of interest for the future as the use of miRNAs in the diagnostics and treatment of UFs is a new and exciting opportunity.

## 1. Introduction

### 1.1. Uterine Fibroids—Overview

Uterine fibroids (UFs) are benign lesions and also the most common neoplasms of the female genital tract worldwide [1,2]. According to various data their prevalence may reach up to 70–80% in predisposed groups of women, but the frequency of the detection depends on selected diagnostic methods [3,4]. The risk factors for the UF’s development include some modifiable and non-modifiable factors [3,5]. According to recent data, the most important ones are: race, age, premenopausal state, hypertension, positive family history, time since last birth and food additives [3].

UFs may cause various clinical issues which might constitute the major reason of the overall deterioration of the quality of life (QoL). Women with UFs report different levels of QoL impairment, from no impact to complete discomfort [6], including sexual life, work performance and relations with partners and family [7]. Heavy menstrual bleeding (HMB), pelvic pressure and pain, dyspareunia, voiding and gastrointestinal problems are among the most frequent clinical symptoms in women with UFs [1,7]. Lesions that cause such symptoms may require a pharmacological or surgical intervention [8,9]. The approximate annual economic burden of UFs on United States healthcare system is extremely high [10]. The highest overall treatment costs are generated in case of patients undergoing hysterectomy due to symptomatic tumors, so the main recommended solution is to reduce the number of such procedures. The lack of understanding about the etiology of UFs contributes to the intensive research of new medical therapies [11]. Nowadays we can observe switch to minimally invasive management which is becoming more common and *is* available in most health care systems [12].

### 1.2. Uterine Fibroids—Overview of Etiology and Pathophysiology

Even with such widespread occurrence of these tumors, the exact mechanisms controlling their development and growth still remain unclear [13,14,15,16]. It is obvious that they are monoclonal tumors arising from the myometrium. UFs develop both from smooth muscle cells and fibroblast components placed in a substantial amount of excessive extracellular matrix (ECM) [15,17].

Multiple studies published to date have identified an important role of estrogen and progesterone in the pathogenesis of those tumors [11,15,18]. Available data suggested that progesterone plays more important role than estrogen in the development and growth of UFs [15,19]. Clinical studies revealed that the proliferation markers in UFs had the highest expression in tissue over the second phase of the cycle [18,19]. UFs contain more sex steroid receptors than normal uterine muscle cells. The main mechanism of action of progesterone is based on the overexpression of cytokine-related genes and the increase of selected growth factor (e.g., transforming growth factor β—TGF-β) concentrations directly in the tumor, which resembles a sort of a self-stimulating process [20,21,22].

### 1.3. Uterine Fibroids—Introduction into Genetics

Development of the whole female genital tract is controlled by the complex interactions of multiple pathways that include gene expression, transcription and epigenetics related to the post-transcriptional regulation and multiple protein translation. In order to achieve and maintain pregnancy, a precise interplay between hormonal signaling in both endometrial and myometrial components must be precisely regulated. Genetic defects are known to be the key points in tumor formation and a great amount of data has recently accumulated in this field. UF’s cells contain multiple gene alterations that differentiate them from normal uterine muscle cells [23]. As in many other cases, a possible functional role of promoter deoxyribonucleic acid (DNA) methylation-mediated gene silencing has been proposed in the pathogenesis of those tumors [24]. However, most of the recent research has highlighted different key pathways and gene expression changes. Mäkinen et al. (2011) were among the first who demonstrated that the mutations in mediator complex subunit 12 gene (*MED12*) were found in the majority of UFs and this finding turned out to be one of the most important changes causing tumor development [25]. These changes may lead to the modification of the transduction of the main signal pathways, e.g., beta-catenin and TGF-β [19,21,26]. Moreover, it has been recently found that *MED12* mutations disrupted mediator kinase activity, implicating altered cyclin function in UFs [27]. Those mutations also dysregulate the canonical wingless-related integration site (Wnt) pathway and the mammalian target of rapamycin (mTOR) signaling pathway which might be associated with autophagy disturbances in UFs [28]. All of these processes may lead to the clonal expansion and tumor growth with abnormal cells remaining sensitive to steroid stimulation. However, the understanding of how genetic modifiers impact multiple UFs development and the related disease severity is still incomplete and require further research [29].

### 1.4. miRNA and Its Biogenesis

Ribonucleic acids (RNAs) are usually classified according to their nucleotide length. Epigenetic events are important gene action modifiers and causes of various human diseases and one of the main mechanisms of such actions is related to various expression of micro-ribonucleic acids (miRNAs). As a result of their potent epigenetic actions, the miRNAs may play a role as diagnostic and therapeutic targets [30]. MiRNAs are non-coding single-stranded RNAs, approximately 22 base pairs long. Among many described functions they are important gene expression regulators [29,31]. Non-coding RNAs, specifically, miRNAs have been demonstrated to play a major role in epigenetic control of gene expression in various organs including the uterus. MiRNAs are transcribed from genes scattered in all chromosomes except the Y chromosome [32].

**Figure 1:**
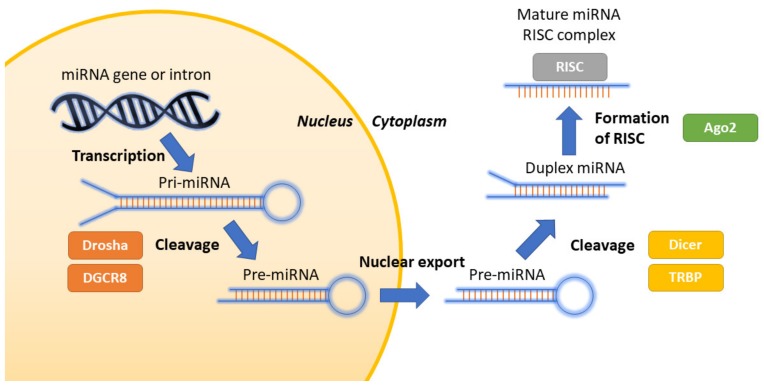
Biogenesis of miRNA.

Half of identified miRNAs are intragenic and processed mostly from introns protein coding genes [33,34]. The main pathway in miRNA biogenesis is the canonical pathway. In this pathway, it all begins in the cell nucleus where the miRNA gene is transcribed by RNA polymerase II/III into primary miRNA—a hairpin loop. In the next step, primary miRNA is cleaved to precursor miRNA, a shorter hairpin loop by a complex containing a ribonuclease III (Drosha) and specific cofactor DiGeorge Syndrome critical region 8 (DGCR8). All other processes take part outside the nucleus as this precursor miRNA molecule is transported to cytoplasm with the use of exportin 5. Precursor miRNA is later processed by endoribonuclease Dicer and the RNA-binding protein (TRBP), into a duplex. Finally, this duplex is separated and forms the mature, single stranded and functional miRNA molecule. 5p or 3p strands of the mature miRNA duplex are incorporated into the Argonaute proteins (AGO) to form a RNA-induced silencing complex (RISC). This complex will be responsible for the translational inhibition either by translational repression or mRNA degradation [33]. Mature miRNAs regulate target gene expression at both transcriptional and translational levels [35,36]. The biogenesis of miRNA is presented in Figure 1.

The non-canonical pathways use different combinations of the proteins involved in the canonical pathway [33]. Importantly, one messenger RNA (mRNA) may be modulated by various miRNAs, resulting in an incredibly complicated regulatory network [29]. What is also of great importance is that various miRNAs might be encoded in a single primary transcript and most of them will be somehow related in function [33,37].

### 1.5. miRNA in Uterine Fibroids—Introduction

Currently available knowledge along with genetic and biochemical evidence supports the essential role of miRNAs in cancer development [30]. One of recent advances in this field is the comprehension of the role of miRNAs in tumorigenesis. An alteration in the level of miRNAs is involved in the formation and growth of several tumors which express a distinct miRNA signature [29,30]. Moreover, abnormal miRNA expression is associated with various female diseases including benign and malignant conditions, as well as fertility disorders [38]. All these findings suggest miRNA-dependent post-transcriptional regulation that appears to play an important role in female reproductive system [29,39,40]. Examples of the abnormal expression of miRNAs were documented in endometriosis and UFs [40]. In addition, numerous genes targeted by the identified miRNAs appeared to be associated with several cancer pathways [41]. In the female genital tract, the vast majority of conducted studies was related to miRNAs role in endometrial cancer. In this malignancy miRNAs were suggested to affect major molecular pathways related to cell proliferation and apoptosis in endometrium. Abnormalities in the function of these miRNA-controlled pathways could cause both benign and malignant conditions such as above-mentioned endometriosis or endometrial cancer [40]. As proposed by Nothnick et al. (2016) [40] miRNAs regulate the actions triggered by female steroids e.g., cell proliferation, apoptosis, ECM formation, matrix metalloproteinases (MMPs) activity or autophagy [40].

The aim of this review is to summarize the current data about the role of miRNAs in the pathophysiology of UFs. In this paper, we also discuss future directions in the miRNA research area like diagnostic opportunities or patient-tailored therapies.

## 2. Methodology

The authors conducted a comprehensive search of peer-reviewed literature published in PubMed of the National Library of Medicine and Google Scholar. The main aim of the searches was to update the information on the biological functions of miRNA and their role in pathophysiology in the context of UF formation and growth. Databases were extensively searched for all original and review articles, as well as book chapters related to UFs and miRNA published in English until March 2020. The following keywords (alone or in combination) were used: uterine fibroid, uterine leiomyoma, microRNA and miRNA. The most relevant articles were reviewed and included as appropriate in this review.

## 3. Discussion

### 3.1. miRNA in Uterine Fibroids—Overview

The discovery of non-coding RNAs has changed the way scientists look into the human genome. Not long ago the existence of miRNAs was obscure and the researchers mainly focused on protein-coding genes [30]. Interestingly, miRNA genes reside between genes, within introns and in a few cases they even reside within exons. Therefore, miRNA may be transcribed either alone or in succession with an mRNA [39]. According to current data, miRNAs play an essential role in the development and differentiation of all tissues including those affected by steroid hormones [42,43].

There is a constant unmet need for the new techniques that would improve the diagnosis and increase the efficacy of therapies in pre- and postmenopausal women with UFs [2,9]. After many years without a real breaking of diagnostic barriers, more attention is now paid to the pathogenesis of UFs, and we are starting to gain better understanding of the molecular genesis of those lesions [44]. Different miRNAs and their abnormal expression were demonstrated in various diseases of the endometrium and myometrium [38,40,45]. Concerning another frequent female genital tract disease, endometriosis, some studies showed that miRNA expression is different between the eutopic and ectopic endometrium and also it is different in women with and without endometriosis [46,47]. According to Laudański et al. (2015), 136 different miRNAs proved to be important in the pathophysiology of this condition in 45 miRNA-based potential pathways [48].

The implication of the menstruation cycle-dependent expression of a large number of genes in healthy myometrium and UF regulation by sex steroids are of great importance. As suggested by Luo and Chegini in 2008 it is still necessary to correlate the expression of the most important miRNAs and their target genes to determine if they are subject to control by those steroids. Such findings would facilitate the performance of new studies concerning the regulatory activity of mechanisms to be used in influencing UF growth or regression [35]. Recent research showed that steroid hormone receptors regulated miRNAs, and, in turn, miRNAs regulated the expression and function of those receptors [42]. Moreover, current data indicate that the expression profile of miRNAs in UF cells differs from the one found in the normal myometrium [49].

### 3.2. miRNA in Uterine Fibroids—Genetic Implications

The first miRNAs, *lin-4* and *let-7*, were both discovered in *Caenorhabditis elegans* [50]. The first transcriptome-miRNA analysis of uterine tissues was conducted in 2007 by Wang et al. [51]. According to the recent studies, results of various UFs may harbor recurrent different gene mutations. Since these mutations are frequently exclusive, apparently multiple other molecular pathways must be involved in these cases. Genetic sequencing and integrative data studies revealed molecularly distinct subtypes of UFs [52,53]. The broad spectrum of chromosomal abnormalities and gene mutations (especially *MED12*, *HMGA1* and *HMGA2*) have been implicated in UF development and growth [54]. Currently available literature described the key importance of *MED12* mutation and high mobility group AT-hook 2 gene (*HMGA2)* alterations as mutually exclusive driver alterations in UFs [52]. According to a study by Je et al. (2012) specific *MED12* exon mutations are consistently associated with UFs and various tumors [55]. The inactivation of *MED12* gene results in the upregulation of TGF-β which may trigger the whole cascade of pathways in UFs [15]. As mentioned before, an increased TGF-β expression in UFs is involved in collagen formation and excessive ECM formation making it one of the major issues in UF biology [21].

*HMGA2* mutation involves a (12;14) chromosomal rearrangement and was observed less frequently than the presence of *MED12* mutation. However, it can be found in almost one fifth of clonally abnormal UFs [29,56]. It is already known that *HMGA2* is a target of miRNA *let-7* family. Exogenous *let-7* miRNAs directly repress the dominant transcript of *HMGA2* and *HMGA2a* [56]. One of the regions of the *HMGA* gene has 7 complementary sites to *hsa-let-7* [57]. In 2018, Mello et al. [57] reported the co-occurrence of *HMGA2* overexpression and *MED12* mutation in UFs. These authors have found that the expression of microRNAs predicted to regulate *HMGA2*. Various miRNAs, such as *hsa-let-7a*, *hsa-miR-26a*, *hsa-miR-26b*, *hsa-miR-93* and *hsa-miR-106b,* were found to be downregulated and negatively correlated with *HMGA2*. These authors have concluded that their finding may explain *HMGA2* overexpression in UFs and could be an important direction of future research [57].

Zavadil et al. (2010) [58] conducted a vast and important study in which they compared global miRNA expression patterns with the expression of their predicted target genes in UFs. These researchers have found that the levels of the most dysregulated miRNAs showed an inverse correlation with the expression levels of predicted target genes in UFs. They have also identified 2674 different mRNAs mRNAs that were significantly dysregulated, with 249 downregulated mRNAs being the targets of 5 upregulated miRNAs (*let-7s*, *miR-21*, *miR-23b*, *miR-27a* and *miR-30a*) and 97 upregulated mRNAs being the targets of 5 downregulated miRNAs (*miR-29b*, *miR-32*, *miR-144*, *miR-197* and *miR-212)* [58].

### 3.3. miRNA and Cell Regulation, Inflammation and Angiogenesis—Potential Pathways in Uterine Fibroids

As described in the previous paragraph the dysregulated genes e.g., *MED12* and *HMGA2* are involved in numerous cellular and molecular functions [58,59]. The expression profiles of miRNAs in UFs and matched myometrium, as well as their isolated smooth muscle cells support the hypothesis that selected miRNAs may target UF-derived genes and are in fact a part of the more complex regulatory networks. Post-transcriptional miRNA-targeted mechanisms are also likely to be involved in the regulation of genes with a complex endocrine, paracrine or autocrine influence by sex steroids, growth factors and cytokines [60]. Various authors assessed miRNA using expression profiling, although only few miRNAs with specific targets were confirmed [58,61,62]. Several tumorigenic pathways seem to be regulated by the most highly dysregulated miRNAs in UFs. A complete list of the biological functions of predicted target genes of miRNAs in UFs was proposed by Zavadil et al. (2010) [58] and covered the following actions:

- mitogen-activated protein kinases (MAPK) signaling,
- gap junction,
- actin skeleton regulation,
- cytokine interaction,
- receptor interaction,
- ECM interaction with different receptors,
- cell cycle regulation,
- calcium signaling regulation,
- Janus kinase (JAK) signaling,
- signal transducer and activator of transcription protein (STAT) regulation,
- muscle hypertrophy,
- peroxisome proliferator-activated receptors (PPAR) regulation,
- nuclear factor kappa-light-chain-enhancer of activated B cells (NF-κB) activation,
- TGF signaling,
- adherens junction,
- Wnt/wingless signaling,
- cell adhesion,
- insulin signaling regulation,
- focal adhesion [58].

**Figure 2:**
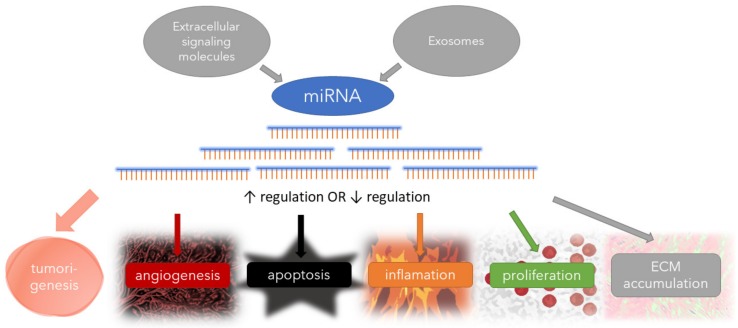
Main molecular events in uterine fibroids (UFs) that involve the regulation by different miRNAs. (extracellular matrix—ECM).

Main biological processes in UFs regulated by miRNAs are presented in Figure 2.

The above-mentioned various control and signal regulations are common in UFs. However, they are also found in many female genital tract malignant tumors allowing them to grow without any host organism control or present an unlimited replicative potential and the ability to invade or metastasize [63].

According to the recently accumulated data, miRNAs are commonly predicted to target genes responsible for ovarian steroid receptors, TGF-β and its receptors and several inflammation-derived genes [29,35,64]. The regulatory functions of miRNAs in inflammation are critical for the initiation and termination of these processes [65,66]. Several miRNAs, e.g., *let-7*, *miR-146* and *miR-155,* were identified to influence the expression of immune response mediators. Inflammation that resulted in the activation of NF-κB-derived pathway has led to the induction of *miR-146*, which, in turn, inhibited the expression of tumor necrosis factor α (TNF-α) receptor-associated factors [65]. Cytokines, which are critical in inflammation have an established influence on UF growth. Some of them, like interleukin (IL) 8, are overexpressed in UFs and exhibit connections with different miRNAs (e.g., *miR-93* or *miR-106b*) [66]. The increased expression of these cytokines might also be associated with HMB due to their activation of coagulation cascades, specifically in women with UFs [35].

Benign and malignant tumors are dependent on angiogenesis for their growth and survival [67]. Hypoxia promotes angiogenesis and according to Hua et al. (2006) [68] it also reduces *miR-15b* and *miR-16* expression. Angiogenesis, being regulated among others by *miR-221* and *miR-222* may indirectly reduce the expression of endothelial nitric oxide synthase (eNOS), a strong angiogenic inducer [68]. According to Mehrad et al. (2007) *miR-18a*, *miR-19a*, *miR-19b* and others to the lesser extent were found to target the expression of connective tissue growth factor (CTGF) and thrombospondin-1 which presents pro-antiangiogenic properties, as well as a dynamic role within the ECM [69].

The above mentioned pathways are important, but not the only ones that may modulate cell regulation, inflammation and angiogenesis of UFs. As the research is ongoing we will for sure receive new interesting data in upcoming years.

#### miRNA and Tumor Senescence

Numerous studies that were conducted to investigate whether cellular senescence is a molecular basis for inactive UFs have demonstrated the trend towards the senescence of UFs which were more pronounced in smaller lesions and in older women. The “older” UFs present high levels of *let-7c*, *let-7d* and *let-7f-2* and a low Ki-67 antigen index [70]. Laser et al. (2010) [70] have suggested that UFs may enter one of two different growth patterns. In the first one the tumor gains additional genetic alterations that promote its growth leading to symptom occurrence and the presence of clinical disease. The second path is the senescence, triggered by molecular and environmental stress factors, with the following influence of several different miRNAs. Apparently, further research targeted into the molecular and environmental factors which cause UF senescence is necessary [70].

### 3.4. Different miRNA Families—A Detailed Description

#### 3.4.1. The *let-7* Family

According to currently available data most members of the *let-7* family are dysregulated in UFs [29,56]. The finding that *let-7* has the effect of the negative regulation of some key target oncogenes like *HMGA2* provides useful information to aid in the understanding of UF pathogenesis [51,71,72]. Interestingly, *let-7* miRNA expression was found to be significantly higher in small UFs, i.e., in masses smaller than 3 cm in maximum diameter [51].

Zavadil et al. (2010) [58] examined the expression of several predicted *let-7* target genes in the ectopically induced presence or absence of *let-7* miRNAs in UF cell lines. The authors found that 65 target genes of *let-7* mRNAs including 45 downregulated that were significantly dysregulated in UFs. Of these selected genes 6 of 11 were found to be repressed by *let-7* miRNAs in UF cells. These authors have concluded that a subset of the predicted target genes may be significantly repressed under experimental conditions, which in turn might be useful information for future studies and treatment implementation [58].

#### 3.4.2. The *miR-15* and *miR-16* Families

These families include the related *miR-15a* and *miR-15b* sequences, and others e.g., *miR-16-1*, *miR-16-2.* However, it is the *miR-15b* which seems the most important in this. Recent studies have shown that *miR-15b* may play a dual role in tumor environment, it may be both the accelerator and blocker [73].

According to a study by Zavadil et al. (2010) [58] *miR-15* and *miR-16* may be associated with different patterns of tumorigenesis of UFs. A detailed analysis of the upregulated targets of the lost *miR-15* and *miR-16* identified their role in important pathways like MAPK, mTOR, JAK, STAT and others [58]. Available data also showed that the dysregulation in *miR-15b* expression levels in UF tissue may modulate cell metabolism and drug resistance [49]. As described previously, miRNAs are also important in angiogenesis, as hypoxia promotes this process and reduces *miR-15b* and *miR-16* expression [68]. A study by Kim et al. (2018) concerning variations in different miRNA expression profiles of UFs demonstrated the upregulated expression level of *miR-15b* in comparison with healthy myometrium [49]. Another study by Guan et al. (2016) [74] demonstrated that *miR-15b* negatively regulated the expression of reversion-inducing cysteine-rich protein with Kazal motifs (RECK) in UFs. These authors have concluded that increased *miR-15b* and decreased RECK expression may contribute to the development of UFs and this pathway might be a new potential target in therapy [74].

#### 3.4.3. The *miR-21* Family

The *miR-21* was one of the first mammalian miRNAs identified. This miRNA has been implicated in an increase in cell proliferation resulting in tumorigenesis and also in tumor fibrosis promotion [75]. It is well-known that abnormal fibrosis and ECM accumulation occur in UFs [17,21] and the abnormal expression of this miRNA was also observed in UFs [29,51]. According to a study by Kang et al. (2012) [76], *miR-21* is induced in TGF-β-dependent vascular smooth muscle cells which in turn may regulate the expression of genes involved in the contraction of smooth muscles [76]. In 2012 Fitzgerald et al. [77] found that UF cells expressed both elevated *miR-21* and a similar pattern of programmed cell death protein 4 (PDCD-4) compared to normal myometrium cells. The knockdown of this miRNA increased PDCD-4 levels, while elevated *miR-21* levels in UFs were related with the decreased PDCD-4 levels. In this regard UFs would differ from other tumors where the loss of PDCD-4 is associated with tumor progression [77].

According to available data, *miR-21* promotes excessive ECM formation by stopping the Smad7 protein which is directly connected with TGF-β pathway [78]. The expression of TGF-β receptor type II is the target of *miR-21* in UFs, so it may mediate its biological activities by binding to TGF-β receptors [29]. In a recent study by Cardozo et al. (2018) [79], the overexpression of *miR-21a-5p* in an immortalized UF was achieved through vector infection. An increased expression of *miR-21* resulted in the increased protein expression of TGF-β3 in UFs and myometrial cells. Numerous important changes were observed in these experiments, e.g., in the expression of fibronectin and collagen genes, changes in genes related to MMP-2, MMP-9 and others. Finally, the *miR-21* upregulation resulted in the increased proliferation of UF cells. These findings emphasize the functional role of this family in UFs formation and growth and could provide the basis for further research [79].

#### 3.4.4. The *miR-29* Family

The importance of the *miR-29* family is one of the best known in UFs [17,80]. The members of this family directly target more than 15 ECM-related genes [81]. As found by Marsh et el. (2016) [80], *miR-29a* levels were decreased in UFs in comparison with normal myometrium. The same group of authors also found that the overexpression of *miRNA-29a* decreased the production of ECM compounds [80].

The behavior of *miR-29b* is relatively similar, as its expression was lower in UF tissue than in the myometrium [51,80]. The results presented recently by Xu et al. (2018) indicated that miRNAs from this family may directly or indirectly regulate many functional genes in protease kinase B (Akt) pathway which, in turn, promotes the survival and growth in response to extracellular signals [82]. Qiang et al. (2014) [83] have demonstrated that the restoration of *miR-29b* inhibited excessive ECM accumulation and UF growth. The authors found that the knockdown of *miR-29b* in myometrial grafts was essential but not sufficient to cause tumorigenesis on its own. Importantly, this study confirmed previous observations that estrogen*s* and progesterone may downregulate miRNA-29b and, as a result, upregulate collagen expression [83].

Another member of the family, *miR-29c* resembles the previous ones, in that it is expressed at lower levels in UFs in comparison with the myometrium [80,84]. Chuang and Khorram (2016) [84] found that *miR-29c* expression was suppressed in UFs, resulting in an increase of the expression of its targets—genes responsible for collagens. This study has also found that this miRNA suppression was mediated through specificity protein 1, NF-κB signaling and epigenetic modulation [84]. Marsh et al. (2016) also reported the decreased protein expression of collagen subtypes I, II and III which was caused by the overexpression of *miRNA-29c* in UF cells [80]. Moreover, also Chuang and Khorram (2019) [85] recently found that the overexpression of *miR-29c* in UFs inhibited the expression of cyclin-dependent kinase 2 (CDK2) protein and mRNA, whereas the knockdown of this miRNA had the opposite effect [85]. Apparently, this particular family of miRNAs, due to its vast actions controlling UFs growth deserves future extensive research.

#### 3.4.5. The *miR-93* and *miR-106* Families

These miRNA families will be described together as the data concerning their function in UFs show some overlapping. The first one, *miR-93,* is a functional RNA and as other miRNAs it is incorporated into RNA-induced silencing complex and has the potential to translational inhibition. Liang et al. (2016) [86] demonstrated that *miR-93* suppressed the apoptosis of gastric cancer cells. The second one, *miR-106b* is a member of the *miR-106b-25* cluster and its role was demonstrated in different cancer types, including breast cancer, gastric cancer and hepatocellular cancer [86]. The role of this type of miRNA has been proven in the repression of multiple members of the Kruppel-like factors [87]. Different studies found that UF expressed significantly lower levels of *miR-93* and that *miR106b* was not differentially expressed in the compared tissues [66]. Chuang et al. (2012) [66] concluded that the differential expression of *miR-93* and *miR-106b* and their regulatory functions in inflammation and tissue changes under the influence of tissue factor 3 (F3) IL-8, CTGF and plasminogen activator inhibitor-1 (PAI-1) may be of importance in UF’s biology [66].

#### 3.4.6. The *miR-146* Family

Before the discovery of the *miRNA-146* family several authors suggested that the pathogenesis of single and multiple UFs might differ. According to a case-control study by Pakiz et al. (2010) women with solitary UFs were mostly healthy, whereas women with multiple UFs had different genotype and were characterized by a positive family history, lower age at menarche, lower parity, younger age at first sexual intercourse and more frequently they were tobacco smokers [88]. Yang et al. (2019) [89] demonstrated that different miRNAs, including *miR-146b-5p*, were dysregulated in women with UFs and played important roles in the development of both solitary and multiple tumors. According to the above-mentioned study long non-coding RNAs (lncRNAs) could influence the expression of miRNAs in the studied group. These authors have concluded that *lnc-AL445665.1-4* might be involved in the development of multiple UFs by interacting with *miR-146b-5p* and that both could be the new potential targets for therapy [89].

#### 3.4.7. The *miR-150* Family

This family of miRNAs was described in gastric cancer where it was thought to promote cell proliferation and was also found to be overexpressed in osteosarcoma [90]. In a study by a Korean group, *miR-150* was found to have an influence on the inhibition of UF growth. In this study the Akt/p27Kip1 pathway was targeted by this miRNA [84]. These authors have found that after transfecting the cells the expression levels of Akt decreased, whereas p27Kip1 significantly increased. Hence, there is a strong evidence that *miR-150* may affect cell cycle regulation in UFs by interaction with the Akt-signaling pathway [91]. Since PI3K/Akt/mTOR is an important intracellular signaling pathway in both normal cell cycle regulation and in UFs development the future research could be directed towards unraveling more details of the described interactions [21].

#### 3.4.8. The *miR-181* and *miR-182* Families

The results of the studies published to date indicate that the *miR-181* family members are highly homologous across different species. The first member of this family, *miR-181a* is recognized as a senescence-associated miRNA and is modulated by reactive oxygen species (ROS) and highly expressed in senescent processes [82]. Recent studies showed that *miR-181a*-targeted genes were related to replicative senescence, cell cycle progression and telomere maintenance [92,93]. It is not the only interesting pathway of this miRNA. According to Wu et al. (2018) [94], *miR-181a* was a potent negative regulator for Akt pathway which may cause cellular senescence observed in monolayer and spheroid cultures. It also exerted its effect on the insulin-like growth factor binding proteins (IGFBP) which might be of some importance in UFs [94].

The second member of this miRNA family has slightly different properties, but it cooperates with *miR-181*. *miR-182* may exert its effect*s* on various pathways, as it is one of miRNAs that are induced in cells responding to stress. Moreover, this miRNA may fine-tune cell cycle in response to damage in selected genes. Previous studies demonstrated that this type of miRNA negatively regulated *BRCA1* expression in different cells [95]. Some recent research also revealed that *miR-182* overexpression induced cell cycle arrest via the inhibition of Akt and forkhead box O 3a (FOXO3a) signaling [96].

#### 3.4.9. The *miR-197* Family

According to currently available data *miR-197* has a confirmed role in human malignancies where it targets key tumorigenic and suppressive genes [97]. Wu et al. (2015) [98] were the first to confirm that *miR-197* was downregulated in human UFs. In the same study *miR-197* was found to inhibit cell proliferation, induce apoptosis and block cells migration in vitro [98]. Another interesting finding of this study was an observation that levonorgestrel could induce *miR-197* expression in UF tissue. This overexpression was suggested to play an important role in cell proliferation and apoptosis modulation [98]. According to the in vitro study of Ling et al. (2015) [99] the downregulation of *miR-197* increased cell growth rate and induced cell cycle arrest in the G0/G1 phase. Conversely, the upregulation of this miRNA was related to the completely opposite effect. It was also found that *miR-197* inhibited cell proliferation by targeting IGFBP5, which was overexpressed in these lesions [99]. In another study by Ling et al. (2015) [100], a different gene expression followed by *miR-197* overexpression was found. These authors have found 17 dominantly dysregulated genes that included genes responsible for tumorigenesis e.g., *DRT7* and *HOXD12* [100]. Kim et al. (2018) [49] have found recently that *miR-197* was able to regulate cell proliferation and suppress tumor development. Moreover, its expression was downregulated in UF cells compared to normal myometrium [49].

#### 3.4.10. The *miR-200* Family

The *miR-200* family consists of 5 members. The majority of *miR-200*s were first found to be involved in cancer pathways as different studies demonstrated their strong suppressive effects on cell transformation, cancer cell proliferation, invasion or metastasis [101]. Zavadil et al. [58] found that *miR-200a* and *miR-200b* were significantly downregulated in the majority of UFs [58]. In particular, *miR-200c* was differentially expressed in UFs and in matched myometrium. In general, UF tumors expressed relatively low levels of *miR-200c* when compared to the healthy surrounding uterine tissue [102,103]. Interestingly, lower levels of *miR-200c* were found in tumors in Afro-American women compared to Caucasian women [102]. Available evidence suggests that the *miR-200* family may mainly function as tumor suppressors, e.g., it activates the NF-κB pathway by phosphorylating its inhibitor [104]. These miRNAs may be induced by ROS and drive UFs into senescence pathway. They may also regulate numerous functional genes in the Akt pathway, similarly to *miR-29b* [82].

According to available data *miR-200c* presents an anti-inflammatory effect in UF cells, as it reduces mRNA and protein levels of pro-inflammatory IL-8 mediator [103]. The same study has found that the knockdown of *miR-200c* caused the opposite effect with an increase in IL-8 mRNA. This effect was mediated through the targeting inhibitor of NF-κB subunit beta (IKBKB) [103]. The role of inflammation in UF pathophysiology is widely accepted [14]. The inflammatory pathways activation is likely to be due to causes other than the reduced expression of IL-8, as some existing studies did not confirm its significant function in the pathophysiology of UFs [14,20]. Interestingly, *miRNA-200c* was also studied in leiomyosarcoma (LMS), where its progressive decline of expression altered the transcriptional regulation of genes controlling the NF-κB pathway, inflammation and cell cycle [105]. *miR-200c* inhibited the expression of CDK2 mRNA in UFs which is similar action to the one described for *miR-29c* [85]. Apparently, the *miR-200* family deserves to be the focus of future studies on new therapies controlling UFs growth and possibly on the differential diagnosis between UFs and LMSs.

#### 3.4.11. Other miRNAs

The above-mentioned miRNAs are known to have a role in the development and growth of the UFs, but they are not the only ones which are differentially expressed in these benign uterine tumors. However, they are those with the best evidence published to date. Currently, there are hundreds of miRNAs that might be related to UFs biology and the data vary depending on authors and centers. In 2012, Georgieva et al. [106] found that even over 50 miRNAs were differentially expressed in UFs when compared to normal myometrium. According to their findings *miR-137*, *miR-217*, *miR-363*, *miR-490* and *miR-4792* were the ones that might have an effect on the biology of UFs [106]. On the other hand, the available data indicate that selected miRNAs such as: *miR-18a*, *miR-20a*, *miR-23a*, *miR-23b*, *miR-26a*, *miR-34a*, *miR-125b*, *miR-139*, *miR-142-5p*, *miR-181a*, *miR-206* and *miR-323* are may be important in UFs biology as they were aberrantly expressed in UFs compared to the normal myometrium [35,107]. In another study by Yoon et al. (2010) [108], *miR-542-3p* was the most significantly upregulated miRNA in UFs. Interestingly, it was also discovered that this miRNA targeted surviving protein, which suppresses cell growth [108].

In a very recent study by Lazzarini et al. (2019) [109], 30 out of 2646 miRNAs presented a significant dysregulation between myometrial progenitor cells and UF progenitor cells [109]. However, only 15 were not close to cut-off values. The Authors separated those miRNAs into downregulating (7 of 2646) and upregulating ones (8 of 2646). Downregulation of miRNAs will involve both ECM and cell junction pathways. The upregulated in the PI3K/Akt signaling pathway, tumorigenesis, cell cycle and cytoskeleton regulation [109]. The role of selected miRNAs in biological pathways of UFs are presented in Table 1.

### 3.5. miRNA and Fibrosis—New Research Targets in UFs

Tissue remodeling is a major process in the progression of fibrotic disorders and the modulation of ECM, adhesion molecules and protease expression are its components. Several studies suggested that miRNAs play an important role in the regulation of ECM accumulation in UFs [17,51]. The TGF-β signaling pathway is one of the most known in fibrogenesis and most of its parts are known to be targeted by one or more miRNAs. In their study on pulmonary fibrosis Kang et al. (2017) [110] found that the regulation of TGF-β signaling molecules by miRNAs appeared to influence the pathogenesis of this illness. They found that TGF-β induced miRNAs such as *miR-21* or *miR-424* that targeted the negative regulators of the TGF-β signaling [110]. Xiao et al. (2015) [111] found that *miR-424* expression was increased by TGF-β and it enhanced the activity of the Smad-dependent signaling pathway. The authors reported that *miR-424* might be involved in myofibroblast differentiation as well [111].

The next miRNA that might be in this field of interest is *miR-101*. Its overexpression in fibroblasts inhibited TGF-β-induced protein and mRNA expression of alpha smooth muscle actin (α-SMA) and different collagens. In the study by Huang et al. (2017) [112] *miR-101* reduced TGF-β-induced contractile activity and stress fiber formation. The authors demonstrated that *miR-101* was an anti-fibrotic miRNA that suppressed the TGF-β-induced activation of fibroblasts by the inhibition of Smad2/3 signaling and target TGF-β receptor I (TGF-βRI) [112]. Another miRNA that may be involved in UF biology is *miR-18a-5p*. In another interesting study Zhang et al. (2017) [113] found that TGF-βRII was a target of *miR-18a-5p*. The authors concluded that *miR-18a-5p* might negatively regulate the TGF-β signaling-mediated epithelial-mesenchymal transition pathway in primary mesenchyme cells by targeting TGF-βRII gene expression [113]. Another miRNA related to TGF-β signaling pathway is *miR-26a*. According to Liang et al. (2014) [114] the overexpression of this miRNA inhibited the nuclear translocation of active Smads which may cause collagen deposition. These data demonstrated that TGF-β modulated the expression of anti-fibrotic *miR-26a* to maintain the pro-fibrotic functions of the TGF-β signaling pathway [114]. New findings indicate that *miR-27b* decreased collagen synthesis and inhibited the expression of α-SMA. This observation may suggest that this miRNA suppresses TGF-β-induced fibroblast activation. Additionally, according to this study, TGF-βRI and Smad2 may be the targets of *miR-27b* [115].

There is also one interesting miRNA which combines both functions—*miR-9-5p*. Current data suggest that some kind of autoregulation occurs between TGF-β and this miRNA. TGF-β may trigger both pro-fibrotic and anti-fibrotic signals. The specific anti-fibrotic signal of *miR-9-5p* is induced by TGF-β and may in turn limit fibrogenesis. The overexpression of *miR-9-5p* in fibroblasts changes myofibroblast metabolism by inhibiting the Smad-dependent TGF-β signaling pathway. Different groups of Smads are modulated in lung fibroblasts with the increased levels of *miR-9-5p.* TGF-βRII was found to be the target of *miR-9-5p* [110,116]. This molecule is another member of various miRNAs that may be related with fibrosis and TGF-β-derived pathway. All of the above-mentioned miRNAs might be of high importance in UFs development and growth.

In the future, different methods used to downregulate the important target genes of upregulated microRNAs may prevent aggressive tumor growth through the repression of the target genes that are responsible for cell signaling. Moreover, ECM formation may play a protective role in avoiding aggressive behaviors of UFs. New studies are required to determine whether miRNAs could be important regulators of single or multiple fibroids formation [58].

### 3.6. miRNAs and Uterine Fibroids—Clinical Implications and Future Directions in Diagnosis and Therapy

#### 3.6.1. miRNAs and the Transformation of a Benign Uterine Fibroid into a Malignant Lesion

Molecular mechanisms that are important for UFs development are still not well understood, although current knowledge on genetics and epigenetics of UFs and LMSs is growing rapidly [15,23]. Available data provide evidence linking several oncogenes and suppressor gene networks with the regulatory function of miRNAs. Some authors hypothesized that the benign component of UF could be a precursor lesion to LMS and some miRNAs were thought to be connected with the cellular changes by targeting protooncogenes and tumor suppressor genes, and the transformation from benign UFs to malignant LMSs [35]. However, an overwhelming amount of data strongly suggests that uterine LMSs are isolated lesions and are not routinely found in association with UFs. A malignant transformation of UFs is extremely rare. Most cases of uterine LMSs are thought to arise de novo. It is also believed that only a small percentage of UFs, with selected histological features and genomic alterations, has the potential for malignant progression [117].The repression of MAPK pathway genes by selected miRNAs may constitute one of the protective mechanisms against fast growth and progression of UFs [58]. The ability of several types of miRNAs, like *miR-20a*, *miR-23a*, *miR-23b*, *miR-26a* and some others mentioned above, to serve as oncogenic or tumor suppressors should be further studied [35].

#### 3.6.2. Different miRNAs in the Differential Diagnosis of Uterine Fibroids and Leiomyosarcomas

Dvorska et al. (2019) [118] found it intriguing that there is no great interest in developing good quality techniques that could differentiate benign UFs and malignant LMSs. The main reason could be related to the very low incidence of these malignant lesions [118]. Nowadays, one of the biggest concerns for surgeons operating on women with UFs is the potential intra-operative fragmentation of LMS during laparoscopic surgery, which may cause intraabdominal spread of malignant cells [119]. The misdiagnosis of LMS with a benign UF may potentially result in a significant morbidity and mortality increase. Therefore, the accurate preoperative diagnosis of uterine masses has become the most important selection criterion for therapy. However, after the Food and Drug Administration communications the awareness in this area has raised substantially. Most women with symptomatic UFs still would prefer minimally invasive procedures, but they would also wish to be certain that the morcellated lesion is not a highly aggressive one [120].

According to available data, clinical manifestations are not useful in distinguishing between such lesions, since both typically present with pain, abnormal uterine bleeding or a diagnosis of a pelvic mass [121]. Imaging differentiation between UFs and LMSs is also challenging due to their potential overlapping features. Magnetic resonance imaging and positron emission tomography have been both so far insufficient [122]. Several biochemical markers may be somehow useful in clinical proceedings. Lactase dehydrogenase (LDH) is one of the possible markers of LMS. The accuracy of LDH elevation although insufficient, is still routinely tested in most cases [123]. Its LDH-D isoenzyme is expressed in patients with uterine sarcomas and the overexpression of LDH-A and LDH-D may act as a potential prognostic biomarker in women with those tumors [124]. In a very recent study, Shalaby et al. (2020) [125] found that survivin is a new possible marker capable of distinguishing between UF and LMS [125].

In this regard, the analysis of different miRNAs serum or urine expression as possible markers of UFs is an attractive opportunity. The activity of miRNAs has been recently assessed in UFs, even if we still do not know the mechanisms of actions of all of them. According to Ravid et al. (2016) [126] the differential miRNA signatures of sarcomas (including LMS) provide novel data with the potential to find diagnostic markers, or therapeutic targets in those lesions [126]. There are also atypical UFs which should be included in this analysis as they might constitute a problem in differential diagnosis [127].

According to a study by Kowalewska et al. (2013) [128], miRNAs were downregulated in malignant compared to benign tissues. In this study *miR-1*, *let-7c*, *let-7f* and *miR-23b* were of different expression in endometrial sarcomas, and *miR-1*, *let-7a*, *let-7b*, *let-7c*, *let-7d*, *let-7e*, *let-7g*, *let-7i*, *miR-133b*, *miR-143*, *miR-214*, *miR-222* in mixed epithelial-mesenchymal tumors. As concluded by these Authors the studied miRNAs might be valuable as possible molecular markers for the differential diagnosis of benign and aggressive uterine tumors [128].

In 2019 Yokoi et al. [129] published data supporting the feasibility of serum circulating miRNAs as potential UFs biomarkers. The authors studied 7 different miRNAs—*miR-191-5p, miR-451a*, *miR-1246*, *miR-4430*, *miR-4485-5p*, *miR-4635*, *miR-6511b-5p*. They have selected 2 of them and reported that the optimal model of *miR-191-5p* and *miR-1246* presented an area under the receiver operating characteristic curve (AUC) for identifying LMS of 0.97 with 95% confidence interval of 0.91–1.00. At the same time, serum LDH had the AUC of only 0.64 and 95% confidence interval of 0.34–0.94 [129]. Earlier in 2017, de Almeida et al. [130] also presented data on miRNAs expression in UFs in differential diagnosis. They did not construct the model as mentioned above, but their data were also of importance. In this study 13 miRNAs presented different expression profiles in UFs and in LMSs compared to the myometrium. However, only 3 miRNAs (*miR-1-3p*, *miR-7-5p*, *miR-140-5p)* showed a significant change in expression in UFs. In LMS a significant overexpression was found for *miR-1-3p*, *miR-7-5p* and *miR-202-3p* [130].

The study by Schiavon et al. (2019) [131] found that expression profiling of *miR-34a-5p, miR-144-3p* and *miR-206* may be useful in distinguishing LMSs from benign uterine tumors. These authors have also found that LMS relapse was related to *miR-148a-3p* overexpression and, in particular, aggressive patterns were related to *miR-124-3p* and *miR-183-5p* [131].

Apparently these interesting findings warrant further research. In our opinion the future diagnostics and differential diagnosis of UFs in women might include the use of molecular biology methods to assess several miRNA patterns.

#### 3.6.3. Investigational Drugs that Target miRNAs in Different Pathological Conditions

In the recent few years, literature regarding the role of miRNAs as important regulators of gene expression in various diseases has rapidly expanded. And the therapeutic potential of miRNAs has gained increasing attention. Some miRNA-based drugs, especially targeted at malignancies have been under observation in different clinical trials. In addition, the use of miRNA in the treatment of the selected non-tumor conditions has been reported, e.g., use in various cardiac diseases [132]. As presented in our review, recent studies in the field of UFs pharmacology reported evidence that drug response and efficacy can be modulated by miRNA-mediated mechanisms. Although symptomatic UFs present multiple treatment challenges, the number of studies that aimed to search for deregulated miRNA/mRNA networks in those tumors is relatively low [29,35,58].

Main strategies that can be used for targeting miRNAs in UFs involve either the inhibition of selected miRNAs actions or the introduction of drugs capable of miRNA functions restoration. The inhibition of miRNAs function has been intended primarily to target oncogenic miRNAs, while the second approach may be regarded as a kind of replacement therapy. The aims of the latter are to deliver and restore the level of suppressors, i.e., specific miRNAs typically downregulated in tumors to restore physiological miRNAs functions [133]. Another hypothetical possibility of the potentially useful miRNA reintroduction is the employment of viral construction containing essential miRNAs coding genes. Although various strategies to reprogram with diverse factors like small molecules, genetic and epigenetic regulators for UFs degeneration exist, there are limitations such as low efficacy, immunogenic problems and unsafe delivery system. The important issues that need to be taken into account when designing miRNA-based therapies include the best miRNA disease-specific target identification, side-effects including toxicity and off-targets drug actions control and efficient and stable drug delivery system [134].

Tiwari et al. (2018) [135] have recently summarized the biology and therapeutic potential of the miRNAs, which are involved in angiogenesis control. Various miRNAs have been found to regulate different stages of angiogenesis in benign tumors such as UFs. All known miRNAs active in angiogenesis can be divided into two groups: angio-miRNAs that promote and angio-miRNAs that inhibit angiogenesis by blocking positive regulators of new blood vessels formation [135]. Examples of the first group include *miR-126*, *miR-130*, *miR-210* and others. The anti-vascular miRNAs target various signaling molecules that stimulate angiogenesis and as a results inhibit capillary and blood vessel formation, the examples are *miR-15b*, *miR-16* or *miR-328* [136].

In summary, miRNAs rapidly became attractive candidates for the development of novel therapeutic approaches in malignant and benign diseases. The knowledge of miRNA pharmacoepigenomics may offer a new fantastic opportunity to develop more effective treatments by providing novel insights into individual variability in drug disposition and response.

#### 3.6.4. miRNA and Uterine Fibroids—Medical Treatment

An important way of thinking about new trends in UF therapy is to discover new drugs as fertility-friendly viable options for non-invasive treatment and the effects that could last for years [137]. Few drugs are strictly directed against UFs, and the majority of the used medications are only used in symptomatic women treatment. Progesterone receptor modulators, gonadotropin-releasing hormone (GnRH) analogs (oral and subcutaneous) or aromatase inhibitors are currently used as non-invasive medical therapies for symptomatic UFs [138]. A long-term, safe and effective treatment is not yet available and several such attempts ended with serious complications [137,138].

The current research idea is to develop a new class of drugs like miRNA inhibitors or miRNA mimics which will offer a new option in the non-invasive treatment and a fight against symptomatic UFs in women [30]. For instance, in their review, Smolle et al. (2017) [139] highlighted the important topic of future directions in miRNAs regulatory role in UFs. Potential usage of miRNA-inhibitors or mimics in clinical practice may constitute promising therapeutic strategies in soft tissue sarcomas that present poor response to chemotherapy [139]. Chuang et al. (2018) [85] concluded that as the downregulation of selected miRNAs in UFs target important cell cycle proteins, potential therapies that could restore these pathways potentially hold the promise of inhibiting UF proliferation and progression [85]. The miRNA expression modulating drugs are apparently difficult to develop and use. The development of UFs and molecular pathways controlling these tumors are dependent on estrogen and progesterone. The main mechanism of action of those hormones (mostly progesterone) in tumorigenesis of UFs is its effect on the increase in the concentration of selected growth factors. Thus, TGF-β has a tremendous effect on the way stem cells are divided and affects their conversion into clonal cells that form UFs [21].

One such example is tranilast, an anti-allergic drug currently used for bronchial asthma and keloid or hypertrophic scars. Tranilast inhibits UF cell proliferation rate and inhibits cell growth and TGF-β-derived collagen biosynthesis [140,141]. Tranilast inhibits the release of inflammatory mediators from mast cells and plays a role in epigenetic regulation [142]. Other studies indicated that it may be used in UF therapy [141], as the effects of tranilast are also related to the *miR-29* family. Tranilast has a direct effect on UF cells through the altered expression of *miR-29c* and genes functionally involved in cell cycle progression and fibrosis [142].

According to Kuokkanen et al. (2010) [143], only 12 miRNAs were significantly upregulated in endometrial biopsies during the late proliferative in comparison with mid-secretory phases. The authors concluded that the changes in miRNAs and cell cycle were related to different progesterone levels in different parts of the phase [143]. It is also an interesting finding that some drugs may have a different potential and actions during different phases, for example due to different receptor expression. However, drugs that inhibit the menstrual cycle should have a stable action during therapy, and not depend on hormones. Available literature includes data concerning *miR-26a* and *miR-181a* which are ones of few miRNAs that directly target the progesterone receptor, and this mechanism could be studied in future experiments [144].

At this point, again, the validity of the concept of the use of co-drugs in UFs should be emphasized. Hopefully, the future research will bring the opportunity to use patient-tailored multivalent therapies with the modulation of selected biochemical or genetic pathways [145]. In our opinion, much of the current research on UFs should focus on which growth factors, cytokines or miRNAs are most frequently associated with specific clinical conditions. For example, anti-proliferative agents are available for UF medical treatment, whereas almost none of the available compounds were introduced specifically as anti-fibrotic ones [17]. As emphasized by Islam et al. (2018) [17] the ECM of UFs plays a critical role in the process of fibrosis and it should be considered as a crucial target for future developments. The miRNA-derived compounds may possess some ability to regulate ECM components and their receptors, as well as UF intracellular signaling pathways [17]. If a possible dependence of UF-specific symptoms on specific molecules, such as growth factors, is confirmed, then optimally selected therapies could be more effective. The synergistic use of different anti-UFs drugs is still under evaluation.

Much research is still needed to discover the complete miRNAs network regulating UF growth, their relationship with ECM and angiogenesis. However, the preliminary results are encouraging and support a strong hope that when it is accomplished, targeting miRNA may be considered as a potential candidate for therapeutic intervention in women with symptomatic UFs.

#### 3.6.5. Uterine Fibroid Location and miRNAs Expression

Kim et al. (2018) [49] presented interesting data on the influence on the occurrence or absence of endometrial cavity distortion of the expression of different miRNAs [49]. In this study the *miR-29* and *miR-200* families were of great importance and were downregulated in UFs. The expression of the progesterone receptor, estrogen receptor and MMPs was upregulated in UF cells in comparison with healthy myometrium. Interestingly, the relative expression of estrogen receptor, MMPs and tissue inhibitors of metalloproteinases (TIMPs) were upregulated in cavity distorting tumors. The authors also found that the expression profile of selected miRNAs in UF cells varied with respect to the occurrence or absence of endometrial cavity distortion. It is new information concerning this topic and the miRNA profile might be involved in the regulation of the biomechanical properties of UF lesions and affect the complete structural and molecular uterine homeostasis [49].

It is still unknown if this is a reason why some UFs might cause infertility, miscarriages or various symptoms, like abnormal bleeding. Subtle changes in the cellular microenvironment that involve miRNAs and their role in chronic inflammation and altered ECM interactions may provide a better explanation of those conditions [109].

#### 3.6.6. Long Non-Coding RNAs

New sequencing methods facilitated the identification of a great number of long non-coding RNAs (lncRNAs), which are built of about 200 nucleotides and represent sense or antisense regions of protein-coding genes. Recent data suggested that similarly to miRNAs, various lncRNAs also had cell- and tissue-specific pattern of expression and may play major regulatory functions in the expression of protein-coding genes [146]. A specific type of lncRNA may upregulate gene transcription through the mediator complex [147].

The role of lncRNAs in tissue fibrosis was studied by Wang et al. (2016) [148]. They found its role in response to activated TGF-β signaling which resulted in the upregulation of different collagens in fibroblasts [148]. The studied lncRNAs presented some regulatory functions through interactions with specific miRNAs and mRNAs. According to a very recent study by Yang et al. (2019) [89] *lnc-AL445665.1-4* is an example of this kind of lncRNA and it is probably involved in the development of multiple UFs by interacting with *miR-146b-5p* [89].

Different lncRNAs exert their function through multiple mechanisms and they were even called the “new player” in the area of ECM modulation and remodeling. The data are still scarce in comparison with miRNAs, but these RNAs might be a new diagnostic, prognostic or predictive biomarker of UFs [149] or be the novel targets for therapy.

**Figure 3:**
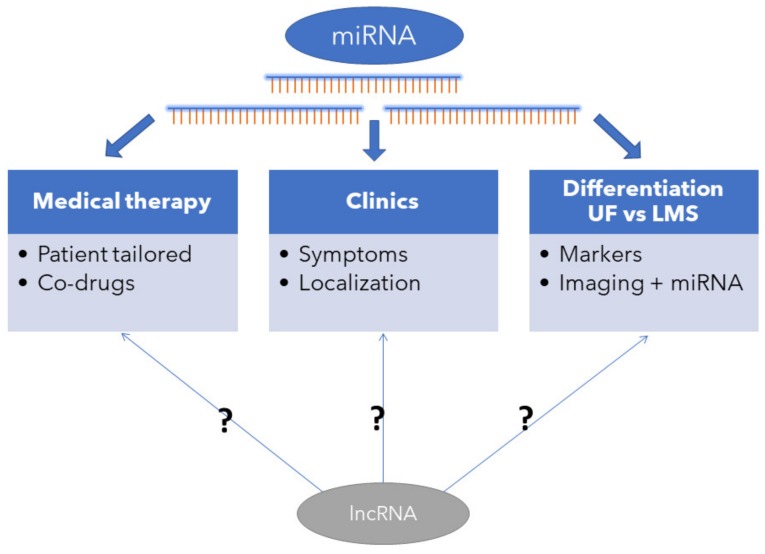
miRNA in women with UFs—possible clinical implications. (unknown role—?)

The summary of possible clinical implications of miRNA in UF management is presented in Figure 3.

## 4. Conclusions

The mechanisms leading to single or multiple UFs development in women are still not fully understood, with various hormonal and genetic regulations thought to be involved. In the current review we have tried to present the role of miRNAs and their different expression profiles in UFs biology. We have also discussed their potential role in UFs cellular signaling, their influence on cell proliferation and ECM accumulation. Numerous questions still remain unanswered. Therefore, more targeted research is needed to understand the complex mechanisms controlling the growth of UFs in order to elucidate role of miRNAs in the tumors. The use of miRNA in the diagnostics and treatment of UFs is another new and exciting opportunity. In light of current limitations in early diagnosis, prevention and medical treatment, an improved understanding of the complex networks of miRNA gene regulation may provide clues for the future development of biomarkers and targeted therapeutics. One of the ideas is to use compounds that modulate UF-derived pathways. Even if miRNA modulating drugs are still unavailable their future clinical utility should be considered. These drugs may provide unique benefits for potential effectiveness alone or when used as co-drugs with others that are already available on the market, e.g., GnRH analogs, aromatase inhibitors or steroid receptor modulators. The idea is to provide therapies that are much more effective, safe and tailored to the patient individual tumor type.

Data related to the regulation of miRNA and its gene targets in the UFs are still insufficient in comparison to those available for malignant tumors. The translational use of miRNA and derived technologies in the clinical care is still at its early phase, but nonetheless it may stimulate new hypotheses about possible new drivers of UFs growth. This area certainly remains an exciting field for the future research in the common female disorder with enormous influence on women’s health status.

## Figures and Tables

**Table 1:** The role of selected miRNAs expression in pathophysiology of UFs.

miRNA Family	Molecular Effects	Overall Clinical Effect
*let-7*	High expression in <3 cm UFs	Tumorigenesis
	HMGA2 negative regulation	
*miR-15*	Angiogenesis regulation	Drug resistance, UF development and growth
	Cell metabolism regulation affecting RECK, MAPK, mTOR, JAK and STAT	
*miR-16*	Angiogenesis regulation	UF development and growth
	Cell metabolism regulation affecting MAPK, mTOR, JAK and STAT	
*miR-21*	Cell proliferation regulation	
	Elevated levels elated with the decreased of PDCD-4 levels	Muscle contraction regulation
	Promotion of excessive ECM formation by stopping the Smad7	UF growth
	Regulation of protein expression of TGF-β3	
*miR-29*	Protease kinase B (Akt) genes regulation	Survival and growth of UF
	ECM accumulation regulation	
	Expression of cyclin-dependent kinase 2 protein and mRNA regulation	
*miR-93 and miR-106*	Regulatory functions in inflammation and tissue changes under the influence of tissue factor 3, interleukin-8, connective tissue growth factor and plasminogen activator inhibitor-1	Survival and growth of UF
*miR-146*	Interactions with long non-coding RNAs	Role in the development of both solitary and multiple tumors
*miR-150*	Akt/p27Kip1 pathway regulation	UF growth
	Cell cycle regulation	
*miR-181 and miR-182*	Senescence-associated miRNAs	UF growth and senescence
	Akt pathway regulation	
	Cell cycle progression and telomere maintenance regulation	
	Cell cycle in response to damage in selected genes regulation	
*miR-197*	Cell proliferation inhibition, apoptosis induction, cell migration block	UF development and growth
	Targeting insulin-like growth factor binding protein 5	
	Tumor development suppression	
*miR-200*	Tumor suppressors	Symptoms and UF development and growth
	Inflammation regulation, e.g., in NF-κB pathway	
	Akt pathway regulation	
	Dysregulated in leiomyosarcoma	

extracellular matrix—ECM; high mobility group proteins—HMGA; Janus kinase—JAK; mitogen-activated protein kinase—MAPK; mammalian target of rapamycin—mTOR; nuclear factor kappa B—NF-κB; programmed cell death protein 4—PDCD-4; reversion-inducing cysteine-rich protein with Kazal motifs—RECK; ribonucleic acid—RNA; signal transducer and activator of transcription protein—STAT; transforming growth factor beta 3—TGF-β3; uterine fibroid—UF.

## Abbreviations

Akt	protease kinase B
AGO	Argonaute protein
AUC	receiver operating characteristic curve
CDK2	cyclin-dependent kinase 2
CTGF	connective tissue growth factor
DGCR8	DiGeorge Syndrome critical region 8
DNA	deoxyribonucleic acid
ECM	extracellular matrix
eNOS	endothelial nitric oxide synthase
F3	tissue factor 3
FOXO3a	forkhead box O 3a
HMB	heavy menstrual bleeding
HMGA	high mobility group AT-hook
IL	interleukin
JAK	Janus kinase
lncRNA	long non-coding RNA
LDH	lactate dehydrogenase
MAPK	mitogen-activated protein kinases
MED12	mediator complex subunit 12
miRNA	microRNA
MMP	matrix metalloproteinase
mRNA	messenger RNA
mTOR	mammalian target of rapamycin
NF-κB	nuclear factor kappa-light-chain-enhancer of activated B cells
PAI-1	plasminogen activator inhibitor-1
PDCD-4	programmed cell death protein 4
PI3K	phosphoinositide 3-kinase
PPAR	peroxisome proliferator-activated receptors
QoL	quality of life
RCEK	reversion-inducing cysteine-rich protein with Kazal motifs
RISC	RNA-induced silencing complex
RNA	ribonucleic acid
ROS	reactive oxygen species
STAT	signal transducer and activator of transcription protein
TGF-β	transforming growth factor beta
TGF-βR	transforming growth factor beta receptor
TNF-α	tumor necrosis factor alpha
UF	uterine fibroid
Wnt	wingless-related integration site
